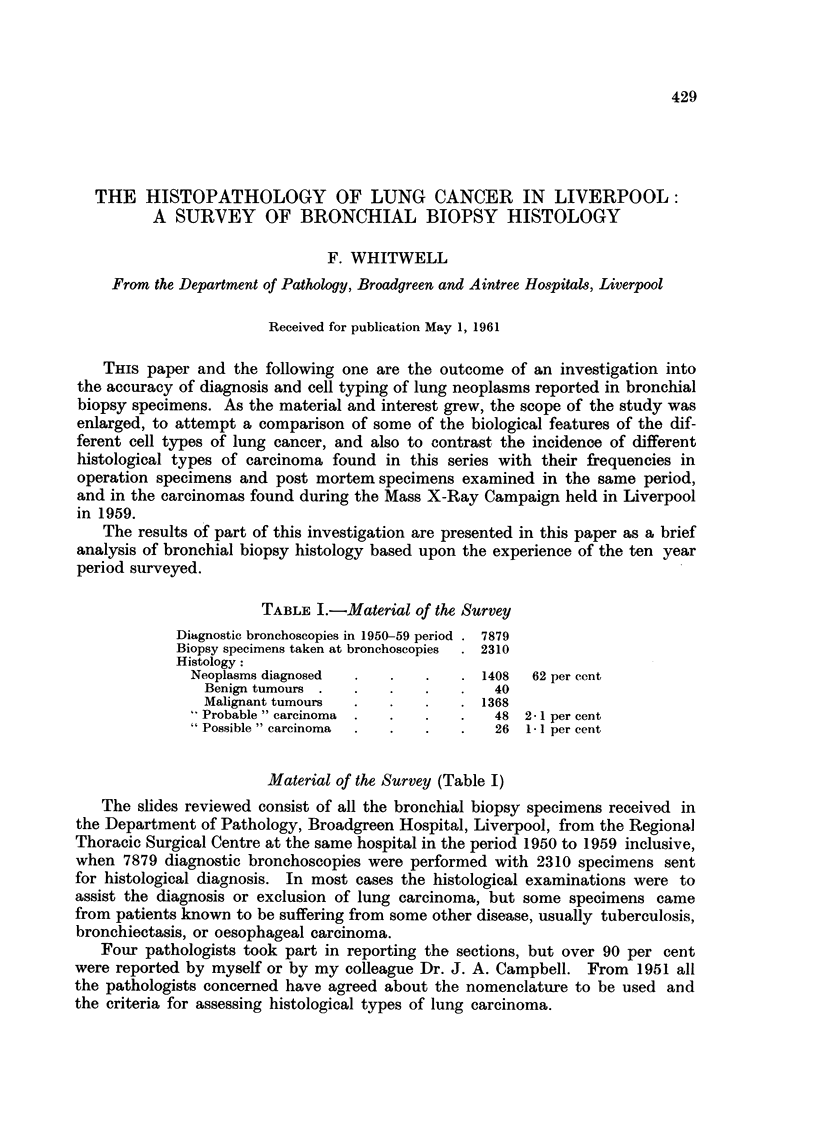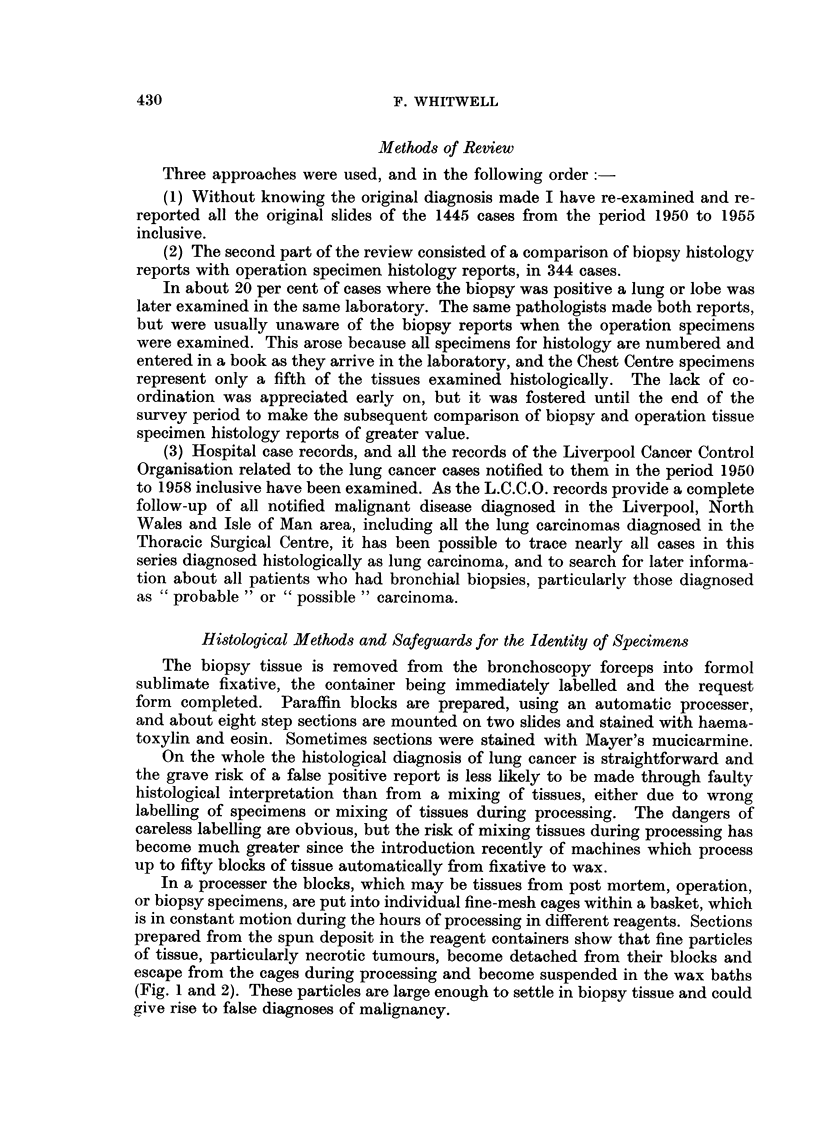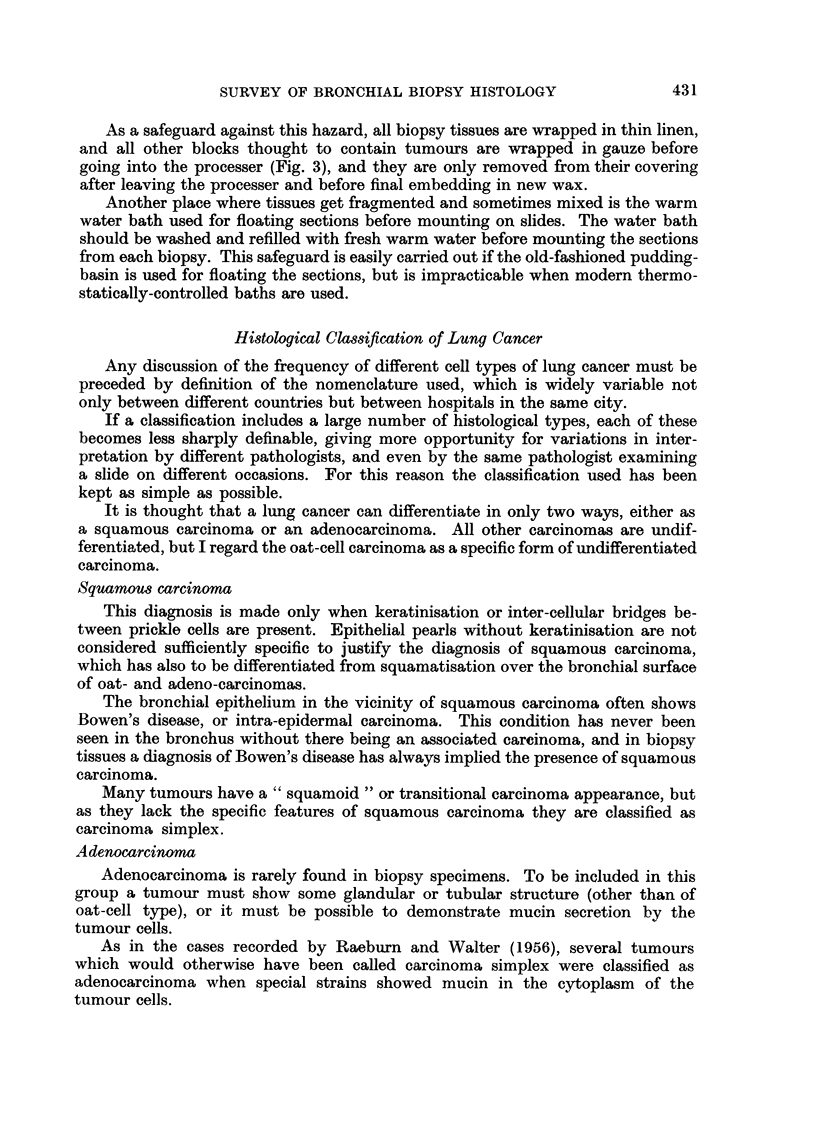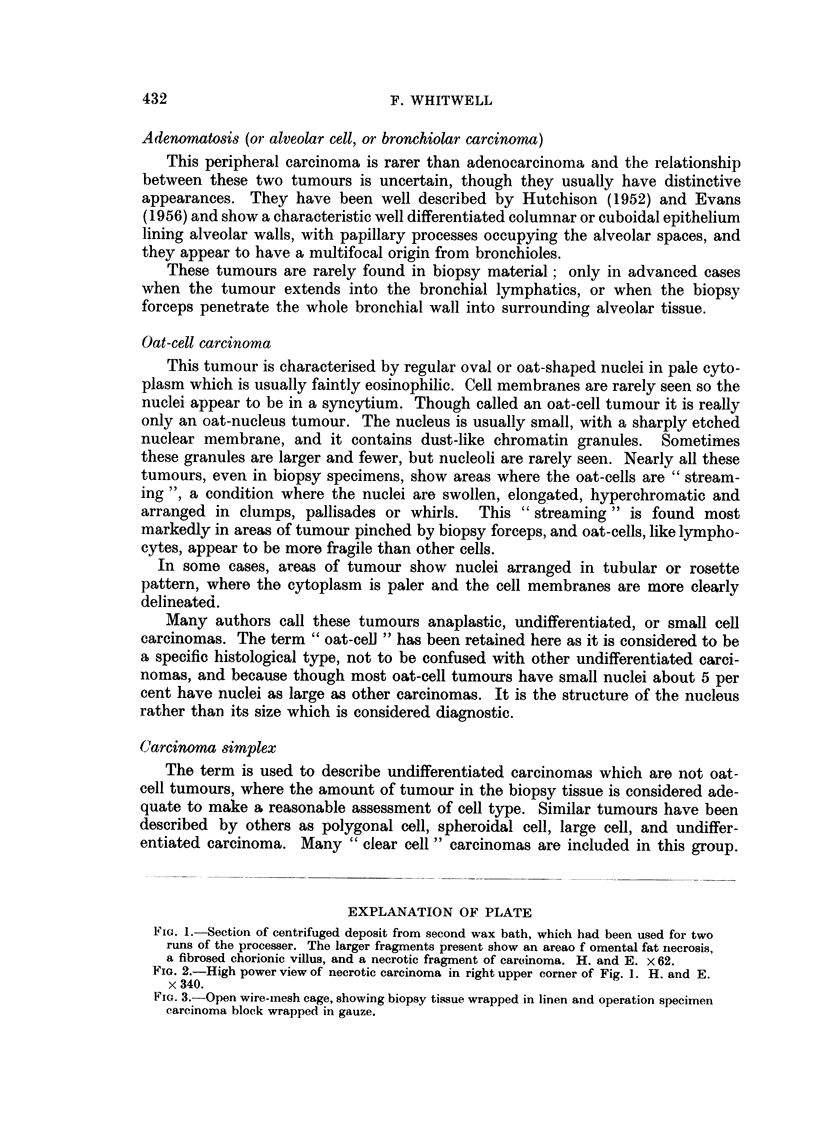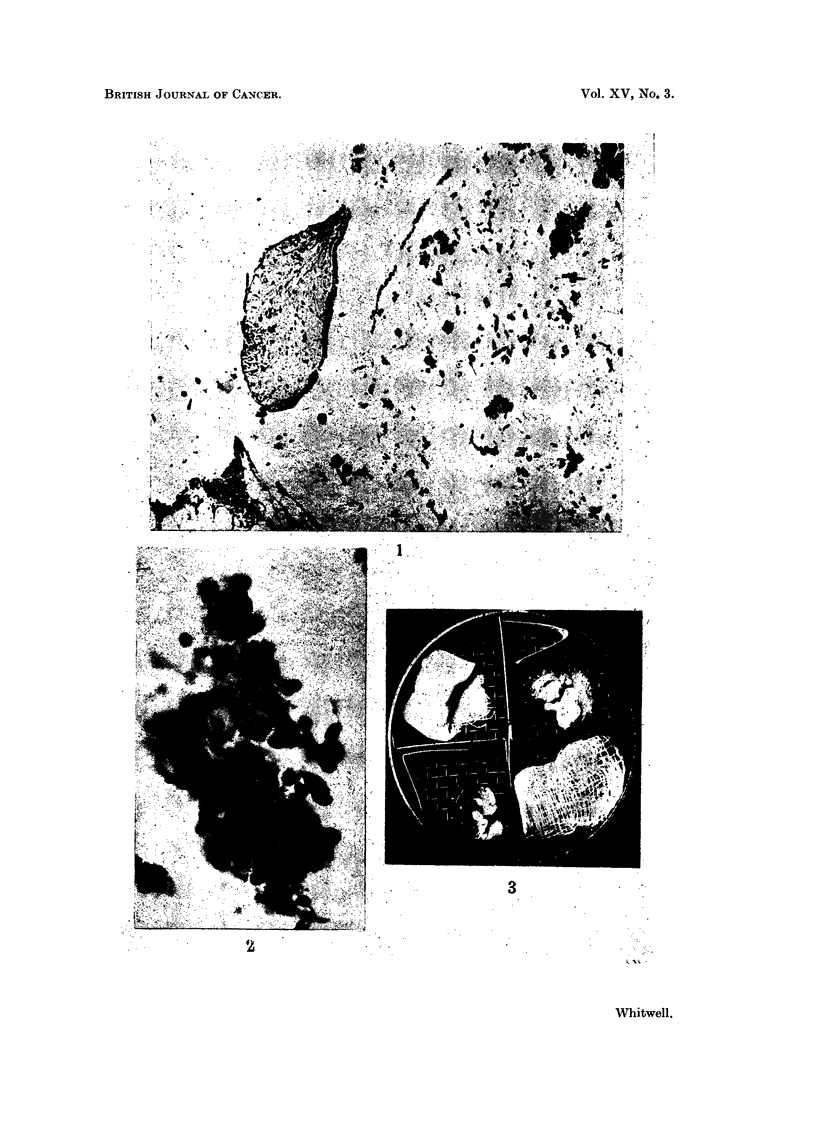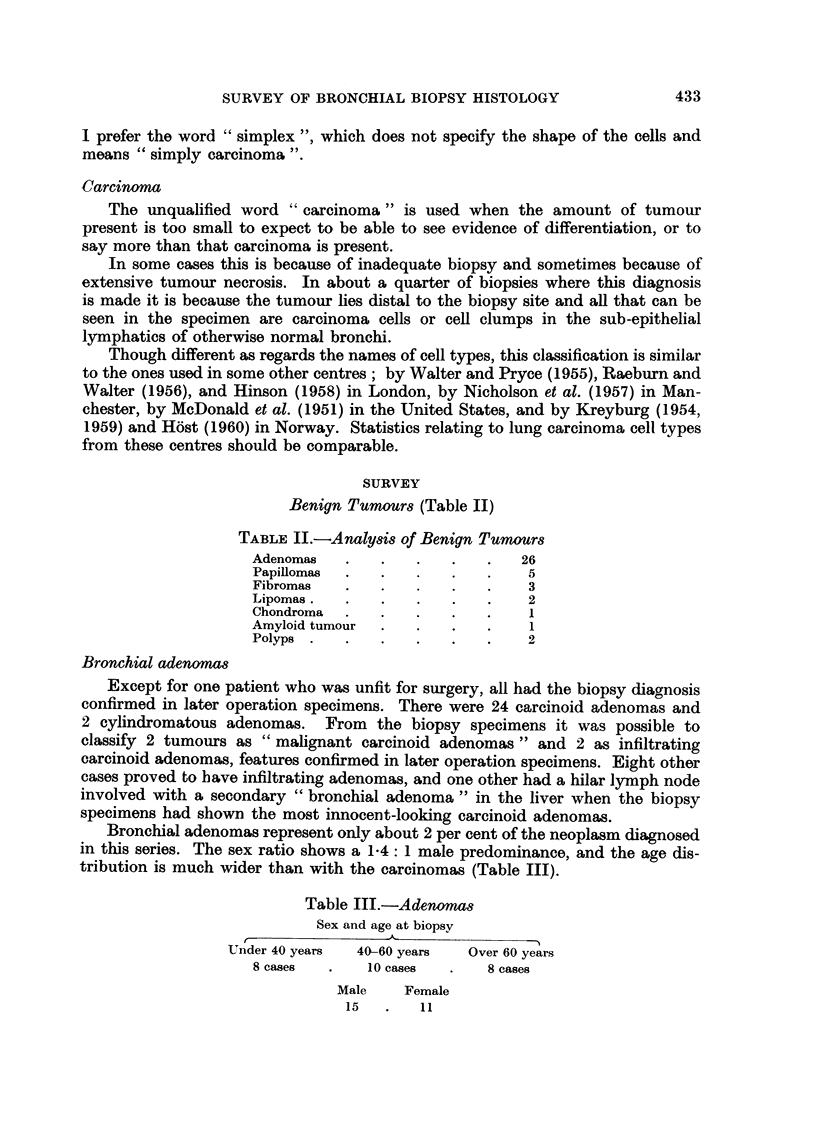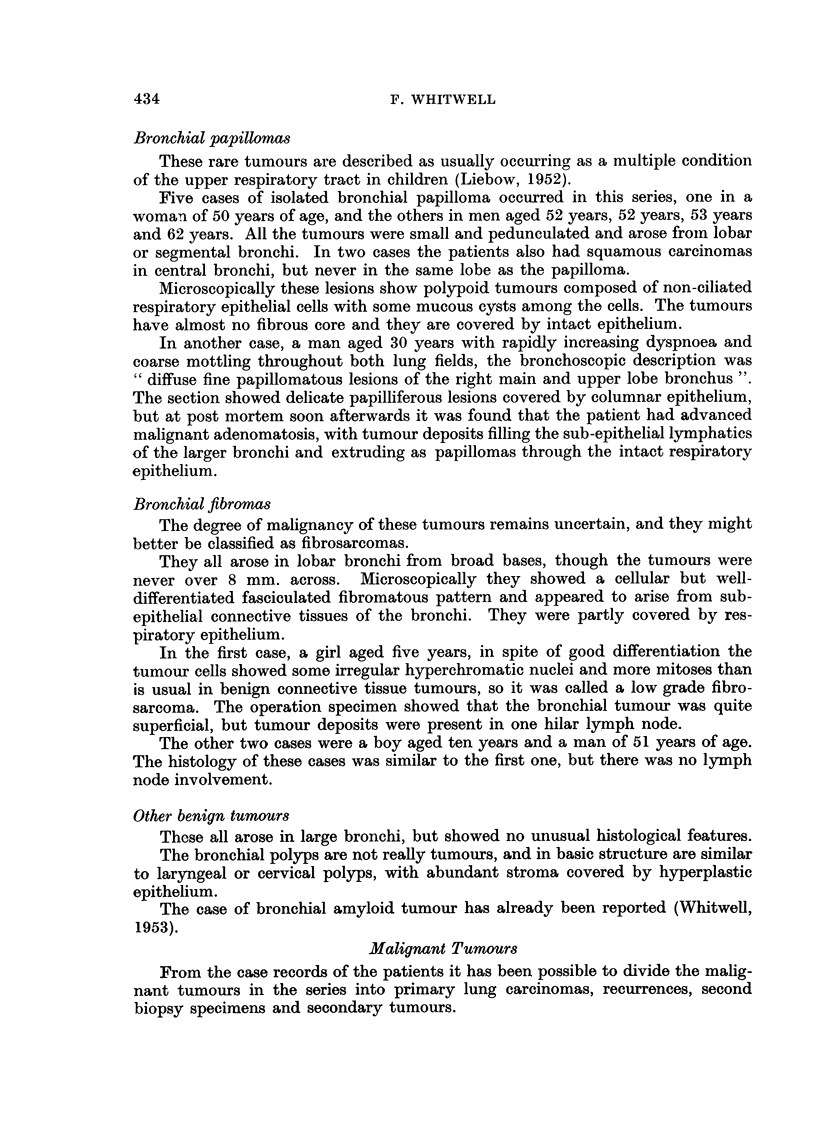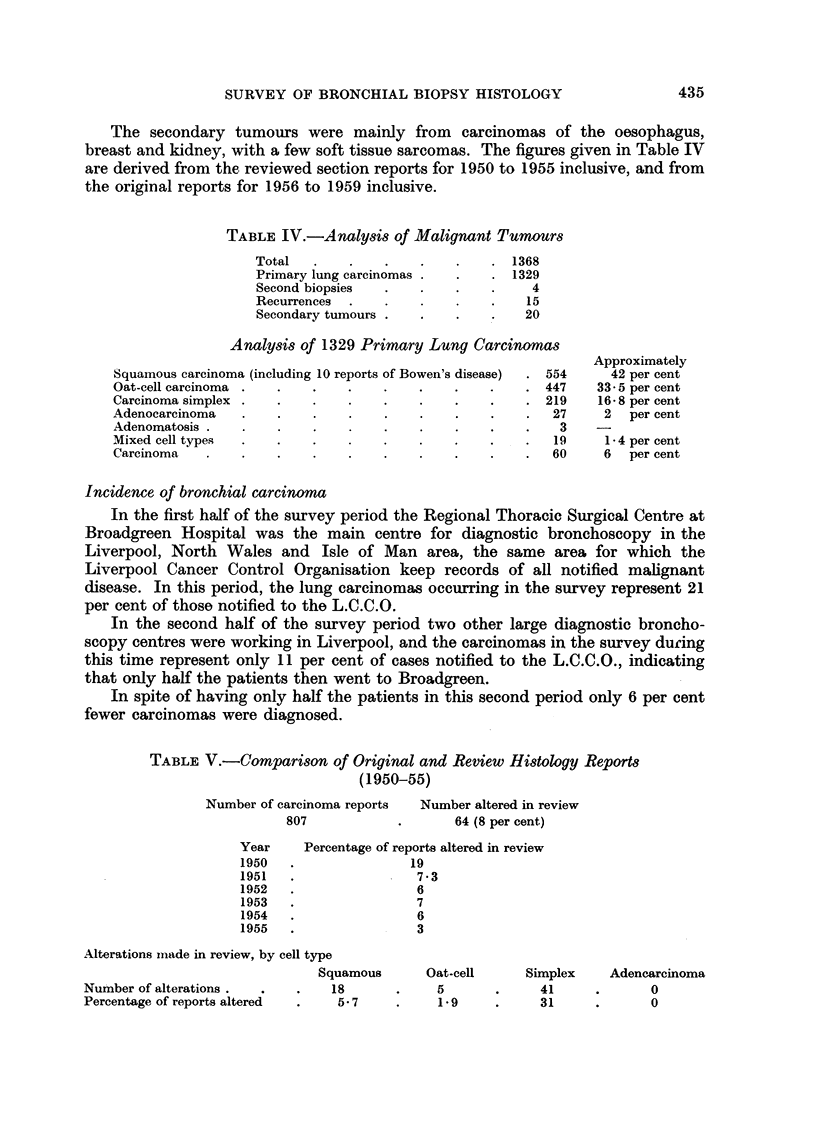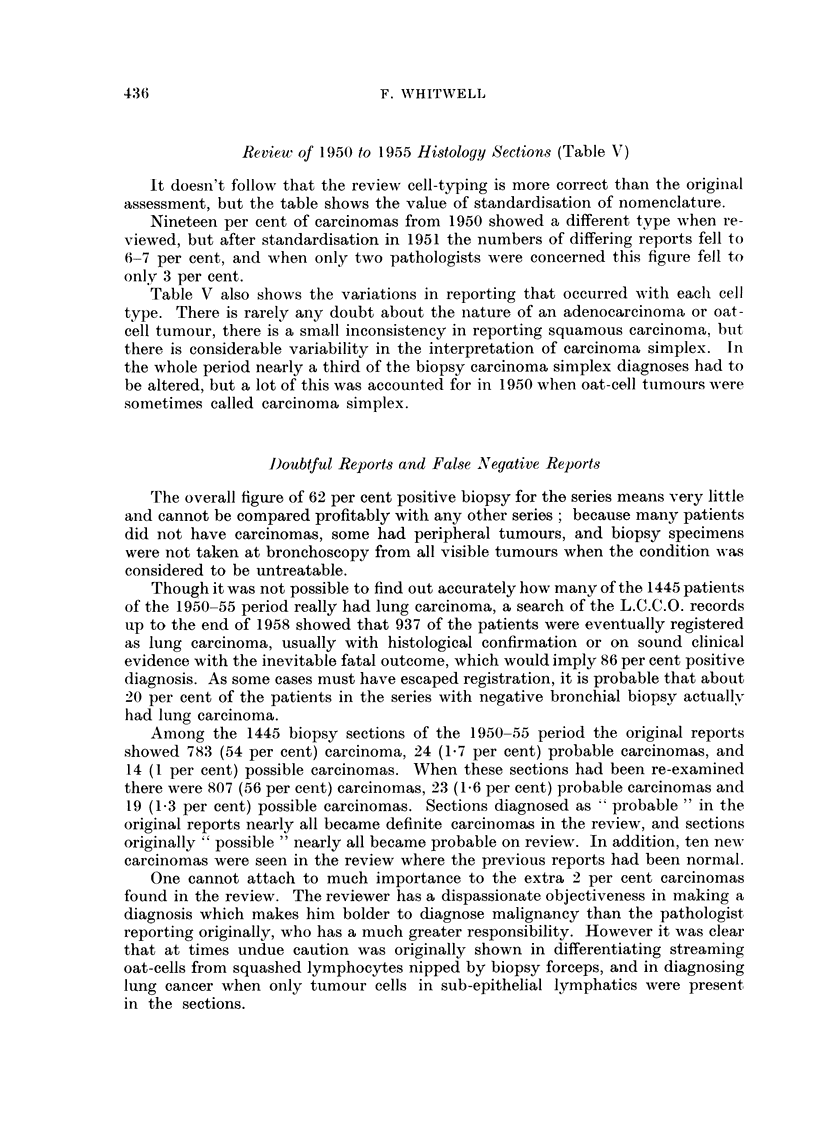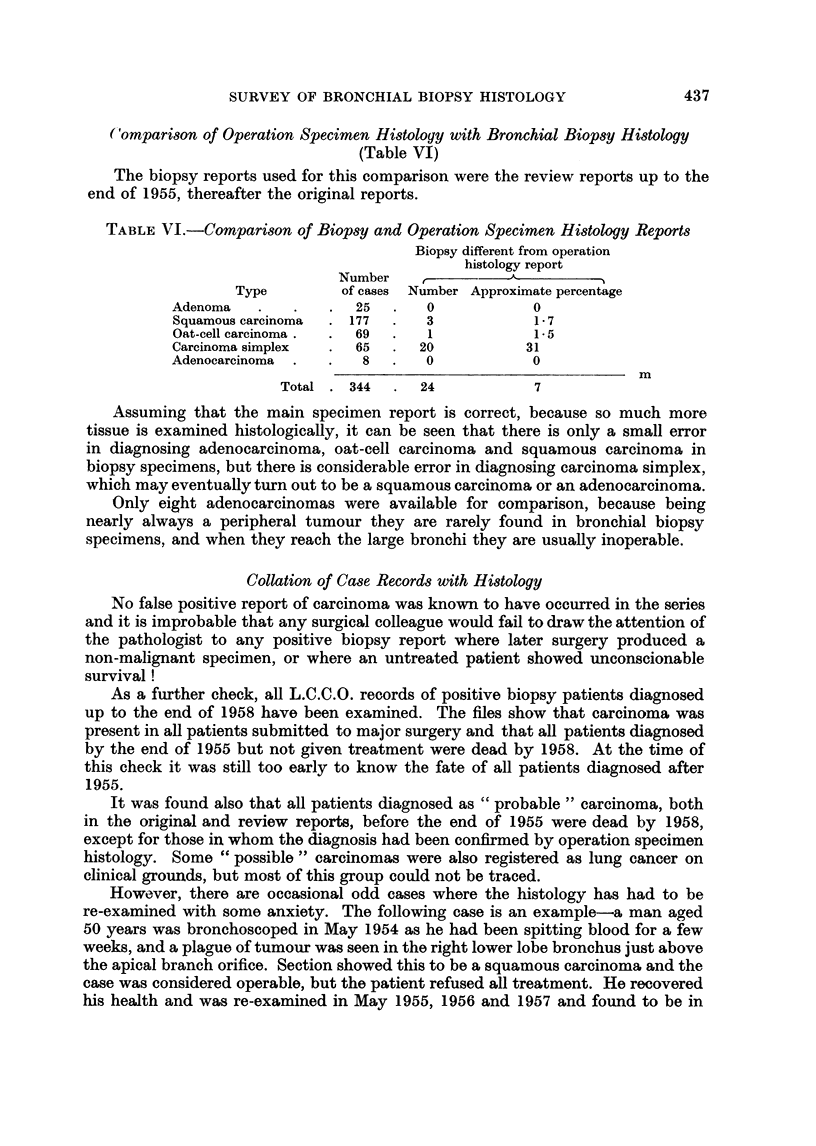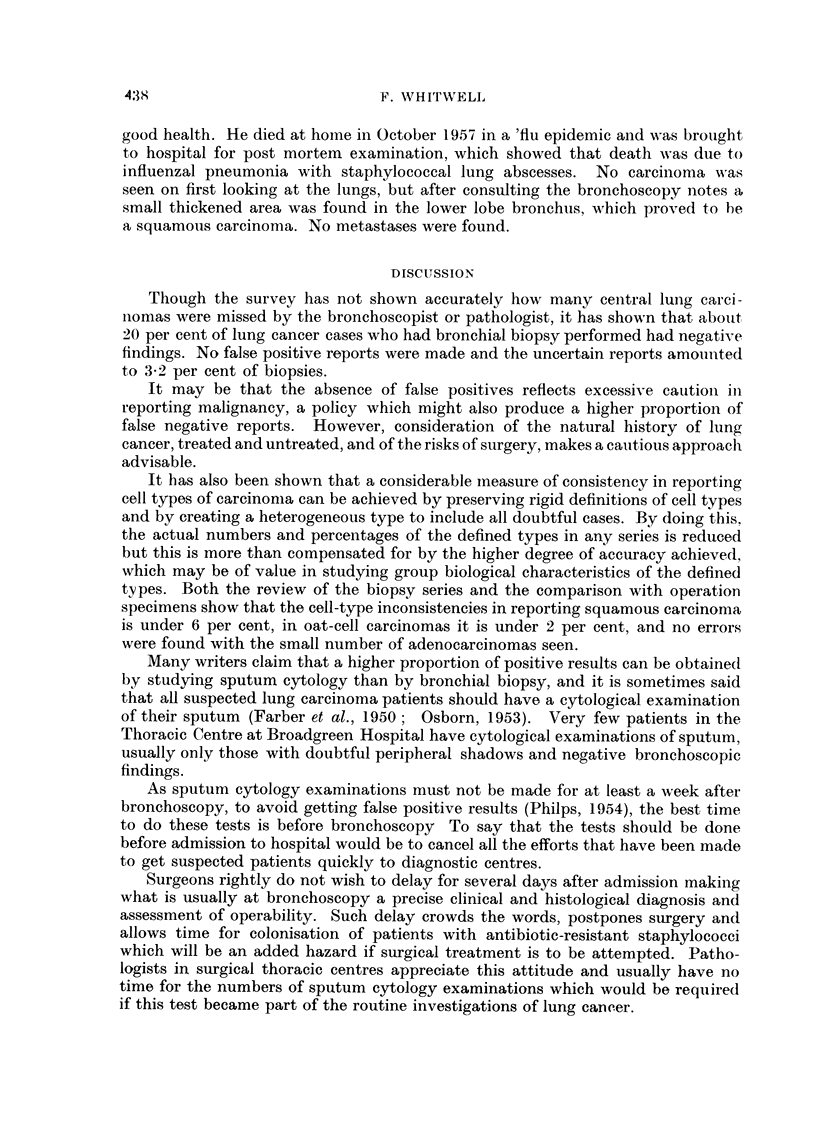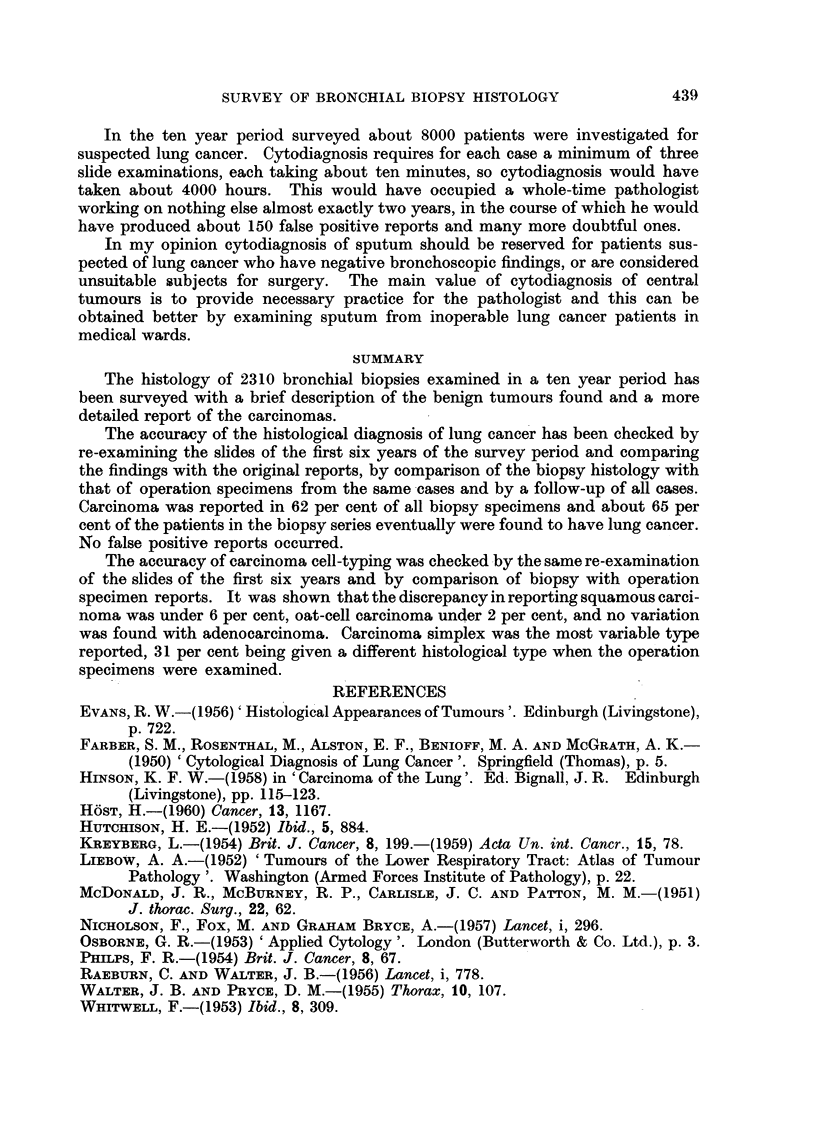# The Histopathology of Lung Cancer in Liverpool: A Survey of Bronchial Biopsy Histology

**DOI:** 10.1038/bjc.1961.53

**Published:** 1961-09

**Authors:** F. Whitwell

## Abstract

**Images:**


### THE HISTOPATHOLOGY OF LUNG CANCER IN LIVERPOOL: A SURVEY OF BRONCHIAL BIOPSY HISTOLOGY

F. WHITWELL

*From the Department of Pathology, Broadgreen and Aintree Hospitals, Liverpool*

Received for publication May 1, 1961

THIS paper and the following one are the outcome of an investigation into the accuracy of diagnosis and cell typing of lung neoplasms reported in bronchial biopsy specimens. As the material and interest grew, the scope of the study was enlarged, to attempt a comparison of some of the biological features of the different cell types of lung cancer, and also to contrast the incidence of different histological types of carcinoma found in this series with their frequencies in operation specimens and post mortem specimens examined in the same period, and in the carcinomas found during the Mass X-Ray Campaign held in Liverpool in 1959.

The results of part of this investigation are presented in this paper as a brief analysis of bronchial biopsy histology based upon the experience of the ten year period surveyed.

TABLE I.—*Material of the Survey*

| | | |
|---|---|---|
| Diagnostic bronchoscopies in 1950–59 period . | 7879 | |
| Biopsy specimens taken at bronchoscopies . | 2310 | |
| Histology : | | |
| Neoplasms diagnosed . . . . . | 1408 | 62 per cent |
| Benign tumours . . . . . . | 40 | |
| Malignant tumours . . . . . | 1368 | |
| " Probable " carcinoma . . . . | 48 | 2·1 per cent |
| " Possible " carcinoma . . . . | 26 | 1·1 per cent |

*Material of the Survey* (Table I)

The slides reviewed consist of all the bronchial biopsy specimens received in the Department of Pathology, Broadgreen Hospital, Liverpool, from the Regional Thoracic Surgical Centre at the same hospital in the period 1950 to 1959 inclusive, when 7879 diagnostic bronchoscopies were performed with 2310 specimens sent for histological diagnosis. In most cases the histological examinations were to assist the diagnosis or exclusion of lung carcinoma, but some specimens came from patients known to be suffering from some other disease, usually tuberculosis, bronchiectasis, or oesophageal carcinoma.

Four pathologists took part in reporting the sections, but over 90 per cent were reported by myself or by my colleague Dr. J. A. Campbell. From 1951 all the pathologists concerned have agreed about the nomenclature to be used and the criteria for assessing histological types of lung carcinoma.

*Methods of Review*

Three approaches were used, and in the following order :—

(1) Without knowing the original diagnosis made I have re-examined and re-reported all the original slides of the 1445 cases from the period 1950 to 1955 inclusive.

(2) The second part of the review consisted of a comparison of biopsy histology reports with operation specimen histology reports, in 344 cases.

In about 20 per cent of cases where the biopsy was positive a lung or lobe was later examined in the same laboratory. The same pathologists made both reports, but were usually unaware of the biopsy reports when the operation specimens were examined. This arose because all specimens for histology are numbered and entered in a book as they arrive in the laboratory, and the Chest Centre specimens represent only a fifth of the tissues examined histologically. The lack of co-ordination was appreciated early on, but it was fostered until the end of the survey period to make the subsequent comparison of biopsy and operation tissue specimen histology reports of greater value.

(3) Hospital case records, and all the records of the Liverpool Cancer Control Organisation related to the lung cancer cases notified to them in the period 1950 to 1958 inclusive have been examined. As the L.C.C.O. records provide a complete follow-up of all notified malignant disease diagnosed in the Liverpool, North Wales and Isle of Man area, including all the lung carcinomas diagnosed in the Thoracic Surgical Centre, it has been possible to trace nearly all cases in this series diagnosed histologically as lung carcinoma, and to search for later information about all patients who had bronchial biopsies, particularly those diagnosed as " probable " or " possible " carcinoma.

*Histological Methods and Safeguards for the Identity of Specimens*

The biopsy tissue is removed from the bronchoscopy forceps into formol sublimate fixative, the container being immediately labelled and the request form completed. Paraffin blocks are prepared, using an automatic processer, and about eight step sections are mounted on two slides and stained with haematoxylin and eosin. Sometimes sections were stained with Mayer's mucicarmine.

On the whole the histological diagnosis of lung cancer is straightforward and the grave risk of a false positive report is less likely to be made through faulty histological interpretation than from a mixing of tissues, either due to wrong labelling of specimens or mixing of tissues during processing. The dangers of careless labelling are obvious, but the risk of mixing tissues during processing has become much greater since the introduction recently of machines which process up to fifty blocks of tissue automatically from fixative to wax.

In a processer the blocks, which may be tissues from post mortem, operation, or biopsy specimens, are put into individual fine-mesh cages within a basket, which is in constant motion during the hours of processing in different reagents. Sections prepared from the spun deposit in the reagent containers show that fine particles of tissue, particularly necrotic tumours, become detached from their blocks and escape from the cages during processing and become suspended in the wax baths (Fig. 1 and 2). These particles are large enough to settle in biopsy tissue and could give rise to false diagnoses of malignancy.

As a safeguard against this hazard, all biopsy tissues are wrapped in thin linen, and all other blocks thought to contain tumours are wrapped in gauze before going into the processer (Fig. 3), and they are only removed from their covering after leaving the processer and before final embedding in new wax.

Another place where tissues get fragmented and sometimes mixed is the warm water bath used for floating sections before mounting on slides. The water bath should be washed and refilled with fresh warm water before mounting the sections from each biopsy. This safeguard is easily carried out if the old-fashioned pudding-basin is used for floating the sections, but is impracticable when modern thermostatically-controlled baths are used.

### *Histological Classification of Lung Cancer*

Any discussion of the frequency of different cell types of lung cancer must be preceded by definition of the nomenclature used, which is widely variable not only between different countries but between hospitals in the same city.

If a classification includes a large number of histological types, each of these becomes less sharply definable, giving more opportunity for variations in interpretation by different pathologists, and even by the same pathologist examining a slide on different occasions. For this reason the classification used has been kept as simple as possible.

It is thought that a lung cancer can differentiate in only two ways, either as a squamous carcinoma or an adenocarcinoma. All other carcinomas are undifferentiated, but I regard the oat-cell carcinoma as a specific form of undifferentiated carcinoma.

### *Squamous carcinoma*

This diagnosis is made only when keratinisation or inter-cellular bridges between prickle cells are present. Epithelial pearls without keratinisation are not considered sufficiently specific to justify the diagnosis of squamous carcinoma, which has also to be differentiated from squamatisation over the bronchial surface of oat- and adeno-carcinomas.

The bronchial epithelium in the vicinity of squamous carcinoma often shows Bowen's disease, or intra-epidermal carcinoma. This condition has never been seen in the bronchus without there being an associated carcinoma, and in biopsy tissues a diagnosis of Bowen's disease has always implied the presence of squamous carcinoma.

Many tumours have a " squamoid " or transitional carcinoma appearance, but as they lack the specific features of squamous carcinoma they are classified as carcinoma simplex.

### *Adenocarcinoma*

Adenocarcinoma is rarely found in biopsy specimens. To be included in this group a tumour must show some glandular or tubular structure (other than of oat-cell type), or it must be possible to demonstrate mucin secretion by the tumour cells.

As in the cases recorded by Raeburn and Walter (1956), several tumours which would otherwise have been called carcinoma simplex were classified as adenocarcinoma when special strains showed mucin in the cytoplasm of the tumour cells.

*Adenomatosis (or alveolar cell, or bronchiolar carcinoma)*

This peripheral carcinoma is rarer than adenocarcinoma and the relationship between these two tumours is uncertain, though they usually have distinctive appearances. They have been well described by Hutchison (1952) and Evans (1956) and show a characteristic well differentiated columnar or cuboidal epithelium lining alveolar walls, with papillary processes occupying the alveolar spaces, and they appear to have a multifocal origin from bronchioles.

These tumours are rarely found in biopsy material ; only in advanced cases when the tumour extends into the bronchial lymphatics, or when the biopsy forceps penetrate the whole bronchial wall into surrounding alveolar tissue.

*Oat-cell carcinoma*

This tumour is characterised by regular oval or oat-shaped nuclei in pale cytoplasm which is usually faintly eosinophilic. Cell membranes are rarely seen so the nuclei appear to be in a syncytium. Though called an oat-cell tumour it is really only an oat-nucleus tumour. The nucleus is usually small, with a sharply etched nuclear membrane, and it contains dust-like chromatin granules. Sometimes these granules are larger and fewer, but nucleoli are rarely seen. Nearly all these tumours, even in biopsy specimens, show areas where the oat-cells are " streaming ", a condition where the nuclei are swollen, elongated, hyperchromatic and arranged in clumps, pallisades or whirls. This " streaming " is found most markedly in areas of tumour pinched by biopsy forceps, and oat-cells, like lymphocytes, appear to be more fragile than other cells.

In some cases, areas of tumour show nuclei arranged in tubular or rosette pattern, where the cytoplasm is paler and the cell membranes are more clearly delineated.

Many authors call these tumours anaplastic, undifferentiated, or small cell carcinomas. The term " oat-cell " has been retained here as it is considered to be a specific histological type, not to be confused with other undifferentiated carcinomas, and because though most oat-cell tumours have small nuclei about 5 per cent have nuclei as large as other carcinomas. It is the structure of the nucleus rather than its size which is considered diagnostic.

*Carcinoma simplex*

The term is used to describe undifferentiated carcinomas which are not oat-cell tumours, where the amount of tumour in the biopsy tissue is considered adequate to make a reasonable assessment of cell type. Similar tumours have been described by others as polygonal cell, spheroidal cell, large cell, and undifferentiated carcinoma. Many " clear cell " carcinomas are included in this group.

---

EXPLANATION OF PLATE

FIG. 1.—Section of centrifuged deposit from second wax bath, which had been used for two runs of the processer. The larger fragments present show an areao f omental fat necrosis, a fibrosed chorionic villus, and a necrotic fragment of carcinoma. H. and E. ×62.

FIG. 2.—High power view of necrotic carcinoma in right upper corner of Fig. 1. H. and E. ×340.

FIG. 3.—Open wire-mesh cage, showing biopsy tissue wrapped in linen and operation specimen carcinoma block wrapped in gauze.

1

2

3

I prefer the word " simplex ", which does not specify the shape of the cells and means " simply carcinoma ".

*Carcinoma*

The unqualified word " carcinoma " is used when the amount of tumour present is too small to expect to be able to see evidence of differentiation, or to say more than that carcinoma is present.

In some cases this is because of inadequate biopsy and sometimes because of extensive tumour necrosis. In about a quarter of biopsies where this diagnosis is made it is because the tumour lies distal to the biopsy site and all that can be seen in the specimen are carcinoma cells or cell clumps in the sub-epithelial lymphatics of otherwise normal bronchi.

Though different as regards the names of cell types, this classification is similar to the ones used in some other centres ; by Walter and Pryce (1955), Raeburn and Walter (1956), and Hinson (1958) in London, by Nicholson *et al.* (1957) in Manchester, by McDonald *et al.* (1951) in the United States, and by Kreyburg (1954, 1959) and Höst (1960) in Norway. Statistics relating to lung carcinoma cell types from these centres should be comparable.

### SURVEY

### *Benign Tumours* (Table II)

TABLE II.—*Analysis of Benign Tumours*

| | |
|---|---|
| Adenomas | 26 |
| Papillomas | 5 |
| Fibromas | 3 |
| Lipomas | 2 |
| Chondroma | 1 |
| Amyloid tumour | 1 |
| Polyps | 2 |

*Bronchial adenomas*

Except for one patient who was unfit for surgery, all had the biopsy diagnosis confirmed in later operation specimens. There were 24 carcinoid adenomas and 2 cylindromatous adenomas. From the biopsy specimens it was possible to classify 2 tumours as " malignant carcinoid adenomas " and 2 as infiltrating carcinoid adenomas, features confirmed in later operation specimens. Eight other cases proved to have infiltrating adenomas, and one other had a hilar lymph node involved with a secondary " bronchial adenoma " in the liver when the biopsy specimens had shown the most innocent-looking carcinoid adenomas.

Bronchial adenomas represent only about 2 per cent of the neoplasm diagnosed in this series. The sex ratio shows a 1·4 : 1 male predominance, and the age distribution is much wider than with the carcinomas (Table III).

Table III.—*Adenomas*

| Sex and age at biopsy | | |
|---|---|---|
| Under 40 years | 40–60 years | Over 60 years |
| 8 cases | 10 cases | 8 cases |

| Male | Female |
|---|---|
| 15 | 11 |

*Bronchial papillomas*

These rare tumours are described as usually occurring as a multiple condition of the upper respiratory tract in children (Liebow, 1952).

Five cases of isolated bronchial papilloma occurred in this series, one in a woman of 50 years of age, and the others in men aged 52 years, 52 years, 53 years and 62 years. All the tumours were small and pedunculated and arose from lobar or segmental bronchi. In two cases the patients also had squamous carcinomas in central bronchi, but never in the same lobe as the papilloma.

Microscopically these lesions show polypoid tumours composed of non-ciliated respiratory epithelial cells with some mucous cysts among the cells. The tumours have almost no fibrous core and they are covered by intact epithelium.

In another case, a man aged 30 years with rapidly increasing dyspnoea and coarse mottling throughout both lung fields, the bronchoscopic description was " diffuse fine papillomatous lesions of the right main and upper lobe bronchus ". The section showed delicate papilliferous lesions covered by columnar epithelium, but at post mortem soon afterwards it was found that the patient had advanced malignant adenomatosis, with tumour deposits filling the sub-epithelial lymphatics of the larger bronchi and extruding as papillomas through the intact respiratory epithelium.

*Bronchial fibromas*

The degree of malignancy of these tumours remains uncertain, and they might better be classified as fibrosarcomas.

They all arose in lobar bronchi from broad bases, though the tumours were never over 8 mm. across. Microscopically they showed a cellular but well-differentiated fasciculated fibromatous pattern and appeared to arise from sub-epithelial connective tissues of the bronchi. They were partly covered by respiratory epithelium.

In the first case, a girl aged five years, in spite of good differentiation the tumour cells showed some irregular hyperchromatic nuclei and more mitoses than is usual in benign connective tissue tumours, so it was called a low grade fibrosarcoma. The operation specimen showed that the bronchial tumour was quite superficial, but tumour deposits were present in one hilar lymph node.

The other two cases were a boy aged ten years and a man of 51 years of age. The histology of these cases was similar to the first one, but there was no lymph node involvement.

*Other benign tumours*

These all arose in large bronchi, but showed no unusual histological features.

The bronchial polyps are not really tumours, and in basic structure are similar to laryngeal or cervical polyps, with abundant stroma covered by hyperplastic epithelium.

The case of bronchial amyloid tumour has already been reported (Whitwell, 1953).

### *Malignant Tumours*

From the case records of the patients it has been possible to divide the malignant tumours in the series into primary lung carcinomas, recurrences, second biopsy specimens and secondary tumours.

The secondary tumours were mainly from carcinomas of the oesophagus, breast and kidney, with a few soft tissue sarcomas. The figures given in Table IV are derived from the reviewed section reports for 1950 to 1955 inclusive, and from the original reports for 1956 to 1959 inclusive.

TABLE IV.—*Analysis of Malignant Tumours*

| | |
|---|---|
| Total | 1368 |
| Primary lung carcinomas | 1329 |
| Second biopsies | 4 |
| Recurrences | 15 |
| Secondary tumours | 20 |

*Analysis of* 1329 *Primary Lung Carcinomas*

| | | Approximately |
|---|---|---|
| Squamous carcinoma (including 10 reports of Bowen's disease) | 554 | 42 per cent |
| Oat-cell carcinoma | 447 | 33·5 per cent |
| Carcinoma simplex | 219 | 16·8 per cent |
| Adenocarcinoma | 27 | 2 per cent |
| Adenomatosis | 3 | — |
| Mixed cell types | 19 | 1·4 per cent |
| Carcinoma | 60 | 6 per cent |

*Incidence of bronchial carcinoma*

In the first half of the survey period the Regional Thoracic Surgical Centre at Broadgreen Hospital was the main centre for diagnostic bronchoscopy in the Liverpool, North Wales and Isle of Man area, the same area for which the Liverpool Cancer Control Organisation keep records of all notified malignant disease. In this period, the lung carcinomas occurring in the survey represent 21 per cent of those notified to the L.C.C.O.

In the second half of the survey period two other large diagnostic bronchoscopy centres were working in Liverpool, and the carcinomas in the survey during this time represent only 11 per cent of cases notified to the L.C.C.O., indicating that only half the patients then went to Broadgreen.

In spite of having only half the patients in this second period only 6 per cent fewer carcinomas were diagnosed.

TABLE V.—*Comparison of Original and Review Histology Reports* (1950–55)

| Number of carcinoma reports | Number altered in review |
|---|---|
| 807 | 64 (8 per cent) |

| Year | Percentage of reports altered in review |
|---|---|
| 1950 | 19 |
| 1951 | 7·3 |
| 1952 | 6 |
| 1953 | 7 |
| 1954 | 6 |
| 1955 | 3 |

Alterations made in review, by cell type

| | Squamous | Oat-cell | Simplex | Adencarcinoma |
|---|---|---|---|---|
| Number of alterations | 18 | 5 | 41 | 0 |
| Percentage of reports altered | 5·7 | 1·9 | 31 | 0 |

### *Review of* 1950 *to* 1955 *Histology Sections* (Table V)

It doesn't follow that the review cell-typing is more correct than the original assessment, but the table shows the value of standardisation of nomenclature.

Nineteen per cent of carcinomas from 1950 showed a different type when reviewed, but after standardisation in 1951 the numbers of differing reports fell to 6–7 per cent, and when only two pathologists were concerned this figure fell to only 3 per cent.

Table V also shows the variations in reporting that occurred with each cell type. There is rarely any doubt about the nature of an adenocarcinoma or oat-cell tumour, there is a small inconsistency in reporting squamous carcinoma, but there is considerable variability in the interpretation of carcinoma simplex. In the whole period nearly a third of the biopsy carcinoma simplex diagnoses had to be altered, but a lot of this was accounted for in 1950 when oat-cell tumours were sometimes called carcinoma simplex.

### *Doubtful Reports and False Negative Reports*

The overall figure of 62 per cent positive biopsy for the series means very little and cannot be compared profitably with any other series ; because many patients did not have carcinomas, some had peripheral tumours, and biopsy specimens were not taken at bronchoscopy from all visible tumours when the condition was considered to be untreatable.

Though it was not possible to find out accurately how many of the 1445 patients of the 1950–55 period really had lung carcinoma, a search of the L.C.C.O. records up to the end of 1958 showed that 937 of the patients were eventually registered as lung carcinoma, usually with histological confirmation or on sound clinical evidence with the inevitable fatal outcome, which would imply 86 per cent positive diagnosis. As some cases must have escaped registration, it is probable that about 20 per cent of the patients in the series with negative bronchial biopsy actually had lung carcinoma.

Among the 1445 biopsy sections of the 1950–55 period the original reports showed 783 (54 per cent) carcinoma, 24 (1·7 per cent) probable carcinomas, and 14 (1 per cent) possible carcinomas. When these sections had been re-examined there were 807 (56 per cent) carcinomas, 23 (1·6 per cent) probable carcinomas and 19 (1·3 per cent) possible carcinomas. Sections diagnosed as " probable " in the original reports nearly all became definite carcinomas in the review, and sections originally " possible " nearly all became probable on review. In addition, ten new carcinomas were seen in the review where the previous reports had been normal.

One cannot attach to much importance to the extra 2 per cent carcinomas found in the review. The reviewer has a dispassionate objectiveness in making a diagnosis which makes him bolder to diagnose malignancy than the pathologist reporting originally, who has a much greater responsibility. However it was clear that at times undue caution was originally shown in differentiating streaming oat-cells from squashed lymphocytes nipped by biopsy forceps, and in diagnosing lung cancer when only tumour cells in sub-epithelial lymphatics were present in the sections.

### *Comparison of Operation Specimen Histology with Bronchial Biopsy Histology* (Table VI)

The biopsy reports used for this comparison were the review reports up to the end of 1955, thereafter the original reports.

TABLE VI.—*Comparison of Biopsy and Operation Specimen Histology Reports*

| Type | Number of cases | Biopsy different from operation histology report: Number | Approximate percentage |
|---|---|---|---|
| Adenoma | 25 | 0 | 0 |
| Squamous carcinoma | 177 | 3 | 1·7 |
| Oat-cell carcinoma | 69 | 1 | 1·5 |
| Carcinoma simplex | 65 | 20 | 31 |
| Adenocarcinoma | 8 | 0 | 0 |
| Total | 344 | 24 | 7 |

Assuming that the main specimen report is correct, because so much more tissue is examined histologically, it can be seen that there is only a small error in diagnosing adenocarcinoma, oat-cell carcinoma and squamous carcinoma in biopsy specimens, but there is considerable error in diagnosing carcinoma simplex, which may eventually turn out to be a squamous carcinoma or an adenocarcinoma.

Only eight adenocarcinomas were available for comparison, because being nearly always a peripheral tumour they are rarely found in bronchial biopsy specimens, and when they reach the large bronchi they are usually inoperable.

### *Collation of Case Records with Histology*

No false positive report of carcinoma was known to have occurred in the series and it is improbable that any surgical colleague would fail to draw the attention of the pathologist to any positive biopsy report where later surgery produced a non-malignant specimen, or where an untreated patient showed unconscionable survival!

As a further check, all L.C.C.O. records of positive biopsy patients diagnosed up to the end of 1958 have been examined. The files show that carcinoma was present in all patients submitted to major surgery and that all patients diagnosed by the end of 1955 but not given treatment were dead by 1958. At the time of this check it was still too early to know the fate of all patients diagnosed after 1955.

It was found also that all patients diagnosed as " probable " carcinoma, both in the original and review reports, before the end of 1955 were dead by 1958, except for those in whom the diagnosis had been confirmed by operation specimen histology. Some " possible " carcinomas were also registered as lung cancer on clinical grounds, but most of this group could not be traced.

However, there are occasional odd cases where the histology has had to be re-examined with some anxiety. The following case is an example—a man aged 50 years was bronchoscoped in May 1954 as he had been spitting blood for a few weeks, and a plague of tumour was seen in the right lower lobe bronchus just above the apical branch orifice. Section showed this to be a squamous carcinoma and the case was considered operable, but the patient refused all treatment. He recovered his health and was re-examined in May 1955, 1956 and 1957 and found to be in good health. He died at home in October 1957 in a 'flu epidemic and was brought to hospital for post mortem examination, which showed that death was due to influenzal pneumonia with staphylococcal lung abscesses. No carcinoma was seen on first looking at the lungs, but after consulting the bronchoscopy notes a small thickened area was found in the lower lobe bronchus, which proved to be a squamous carcinoma. No metastases were found.

### DISCUSSION

Though the survey has not shown accurately how many central lung carcinomas were missed by the bronchoscopist or pathologist, it has shown that about 20 per cent of lung cancer cases who had bronchial biopsy performed had negative findings. No false positive reports were made and the uncertain reports amounted to 3·2 per cent of biopsies.

It may be that the absence of false positives reflects excessive caution in reporting malignancy, a policy which might also produce a higher proportion of false negative reports. However, consideration of the natural history of lung cancer, treated and untreated, and of the risks of surgery, makes a cautious approach advisable.

It has also been shown that a considerable measure of consistency in reporting cell types of carcinoma can be achieved by preserving rigid definitions of cell types and by creating a heterogeneous type to include all doubtful cases. By doing this, the actual numbers and percentages of the defined types in any series is reduced but this is more than compensated for by the higher degree of accuracy achieved, which may be of value in studying group biological characteristics of the defined types. Both the review of the biopsy series and the comparison with operation specimens show that the cell-type inconsistencies in reporting squamous carcinoma is under 6 per cent, in oat-cell carcinomas it is under 2 per cent, and no errors were found with the small number of adenocarcinomas seen.

Many writers claim that a higher proportion of positive results can be obtained by studying sputum cytology than by bronchial biopsy, and it is sometimes said that all suspected lung carcinoma patients should have a cytological examination of their sputum (Farber *et al.*, 1950 ; Osborn, 1953). Very few patients in the Thoracic Centre at Broadgreen Hospital have cytological examinations of sputum, usually only those with doubtful peripheral shadows and negative bronchoscopic findings.

As sputum cytology examinations must not be made for at least a week after bronchoscopy, to avoid getting false positive results (Philps, 1954), the best time to do these tests is before bronchoscopy To say that the tests should be done before admission to hospital would be to cancel all the efforts that have been made to get suspected patients quickly to diagnostic centres.

Surgeons rightly do not wish to delay for several days after admission making what is usually at bronchoscopy a precise clinical and histological diagnosis and assessment of operability. Such delay crowds the words, postpones surgery and allows time for colonisation of patients with antibiotic-resistant staphylococci which will be an added hazard if surgical treatment is to be attempted. Pathologists in surgical thoracic centres appreciate this attitude and usually have no time for the numbers of sputum cytology examinations which would be required if this test became part of the routine investigations of lung cancer.

In the ten year period surveyed about 8000 patients were investigated for suspected lung cancer. Cytodiagnosis requires for each case a minimum of three slide examinations, each taking about ten minutes, so cytodiagnosis would have taken about 4000 hours. This would have occupied a whole-time pathologist working on nothing else almost exactly two years, in the course of which he would have produced about 150 false positive reports and many more doubtful ones.

In my opinion cytodiagnosis of sputum should be reserved for patients suspected of lung cancer who have negative bronchoscopic findings, or are considered unsuitable subjects for surgery. The main value of cytodiagnosis of central tumours is to provide necessary practice for the pathologist and this can be obtained better by examining sputum from inoperable lung cancer patients in medical wards.

### SUMMARY

The histology of 2310 bronchial biopsies examined in a ten year period has been surveyed with a brief description of the benign tumours found and a more detailed report of the carcinomas.

The accuracy of the histological diagnosis of lung cancer has been checked by re-examining the slides of the first six years of the survey period and comparing the findings with the original reports, by comparison of the biopsy histology with that of operation specimens from the same cases and by a follow-up of all cases. Carcinoma was reported in 62 per cent of all biopsy specimens and about 65 per cent of the patients in the biopsy series eventually were found to have lung cancer. No false positive reports occurred.

The accuracy of carcinoma cell-typing was checked by the same re-examination of the slides of the first six years and by comparison of biopsy with operation specimen reports. It was shown that the discrepancy in reporting squamous carcinoma was under 6 per cent, oat-cell carcinoma under 2 per cent, and no variation was found with adenocarcinoma. Carcinoma simplex was the most variable type reported, 31 per cent being given a different histological type when the operation specimens were examined.

### REFERENCES

Evans, R. W.—(1956) 'Histological Appearances of Tumours'. Edinburgh (Livingstone), p. 722.

Farber, S. M., Rosenthal, M., Alston, E. F., Benioff, M. A. and McGrath, A. K.—(1950) 'Cytological Diagnosis of Lung Cancer'. Springfield (Thomas), p. 5.

Hinson, K. F. W.—(1958) in 'Carcinoma of the Lung'. Ed. Bignall, J. R. Edinburgh (Livingstone), pp. 115–123.

Höst, H.—(1960) *Cancer*, **13**, 1167.

Hutchison, H. E.—(1952) *Ibid.*, **5**, 884.

Kreyberg, L.—(1954) *Brit. J. Cancer*, **8**, 199.—(1959) *Acta Un. int. Cancr.*, **15**, 78.

Liebow, A. A.—(1952) 'Tumours of the Lower Respiratory Tract: Atlas of Tumour Pathology'. Washington (Armed Forces Institute of Pathology), p. 22.

McDonald, J. R., McBurney, R. P., Carlisle, J. C. and Patton, M. M.—(1951) *J. thorac. Surg.*, **22**, 62.

Nicholson, F., Fox, M. and Graham Bryce, A.—(1957) *Lancet*, i, 296.

Osborne, G. R.—(1953) 'Applied Cytology'. London (Butterworth & Co. Ltd.), p. 3.

Philps, F. R.—(1954) *Brit. J. Cancer*, **8**, 67.

Raeburn, C. and Walter, J. B.—(1956) *Lancet*, i, 778.

Walter, J. B. and Pryce, D. M.—(1955) *Thorax*, **10**, 107.

Whitwell, F.—(1953) *Ibid.*, **8**, 309.